# American Heart Association’s Behavioral Roundtable for Preventable Disparities

**Published:** 2009-03-15

**Authors:** Judith Wylie-Rosett, Njeri Karanja

**Affiliations:** Albert Einstein College of Medicine, Department of Epidemiology and Population Health; Kaiser Permanente Northwest, Portland, Oregon

## Introduction

The American Heart Association (AHA) developed a behavioral roundtable to address cardiovascular disease (CVD) disparities, with a focus on the primary prevention of obesity. This roundtable considered the preventable differences in the indicators of health of different population groups, often defined by race, ethnicity, sex, educational level, socioeconomic status, and geographic location of residence. To reduce the rates of CVD in disproportionately affected population groups, we explored behavioral strategies for each of the 5 risk stages: 1) no known cardiovascular risk, 2) known cardiovascular risk, 3) acute CVD, 4) rehabilitation, and 5) chronic CVD. We examined sample AHA programs targeting each of the risk stages to consider how to pose questions about reach, efficacy/effectiveness, adoption, implementation, and maintenance (the RE-AIM evaluation framework). The strategies outlined in this article can be used to develop collaborations for planning, implementing, and evaluating possible interventions to reduce CVD disparities.

## Disparate CVD Rates: The Rationale for Identifying Special Populations

The AHA Nutrition Committee, with support from its Industry Nutrition Advisory Panel (INAP), established several behavioral roundtables to address behavioral issues and translation to practice focusing on the AHA mission. Each roundtable, with representatives from the Nutrition Committee, INAP, and AHA staff, brainstormed ideas to help the Nutrition Committee integrate behavioral issues into strategic and program planning. The special population behavioral roundtable, which focused on behavioral issues for populations with disparate rates of cardiovascular risk, included 2 Nutrition Committee members who became the authors of the report. Using an iterative process, we examined potential applications of the roundtable's recommendations to existing AHA programs. Our goal was to provide a framework to help AHA staff and volunteers at the national and affiliate levels as they plan and implement AHA programs that address the needs of special population groups. This article can also help other agencies as they implement AHA programs and focus on reducing health disparities.

Our roundtable focused on preventable differences in the widening gap in CVD morbidity and mortality (1,2). To explore how to develop behavioral strategies for reducing the disparate rates of CVD in special population groups, we examined CVD risk stages and needs among people who may have 1) no known or identified cardiovascular risk factors (possibly with undiagnosed CVD risk factors), 2) known or identified risk factors (eg, obesity, diabetes, hypertension), 3) acute CVD problems, 4) rehabilitation, or 5) chronic CVD problems and a need for secondary prevention.

The Institute of Medicine's multilevel approach (3) provides a framework for examining the environment in relation to risk using concentric circles starting with the individual and moving outward to the family, community, and society. Using this multilevel approach is helpful in examining how individual socioeconomic and racial/ethnic status link to health and social systems as determinants of individual and population health (3). Because of the current obesity epidemic, much of our roundtable discussion addressed environmental issues and policies that may promote obesity or leanness (4-6). A recent statement from the AHA emphasized the need for population-based strategies that target the social and physical environment as a means to promote healthy eating and physical activity (6). Our roundtable considered infrastructures that are associated with socioeconomic status and with low-income families living in increasingly obesogenic environments, and we discussed the importance of using techniques that examine the interrelationship among variables associated with poverty (7-9). Although food insecurity has been associated with a 2-fold greater risk of obesity, these differences can also be accounted for by differences in education, income, race/ethnicity, marital status, and general health (9). We considered lack of transportation, safety concerns, care responsibilities, and the availability of parent/adult volunteer coaches. We noted poverty's effects on food-purchasing habits, for example, that low-cost options in grocery stores are often limited. Of particular concern was the cost difference between fresh vegetables and fruits and more highly processed foods with added sugar and fat (8).

The variables that we thought were important in addressing special population needs at each risk stage are listed in the Appendix. For the earlier stages, we considered 1) environmental factors that focused on different rates of access and economic opportunity and 2) psychosocial factors that focused on cultural and individual beliefs in relation to life experiences and competing priorities. Our consideration of more advanced risk stages focused on health care systems as well as factors that influence people at high risk of CVD within vulnerable population groups.

## CVD Disparity and Program Evaluation Focusing on Special Population Groups

We considered the importance of community partnerships — in which community representatives participate in defining research problems, interpreting data, and applying findings — in fostering community-based participatory research methods for the evaluation of disparate rates of CVD or its risk factors (10,11). Our discussion of evaluation focused on the RE-AIM framework (12), which is well suited to the community-based participatory research approach that focuses on disparity. The components and evaluation target includes Reach (What proportion of the target population participated in the intervention?), Efficacy/Effectiveness (What is the success rate if implemented according to the plan or protocol?), Adoption (What proportion of community organizations, classes/schools, and practices opted for the intervention?), Implementation (To what extent is the intervention implemented as intended in the real-world setting?), and Maintenance (To what extent is the program sustainable over time?). We discussed how the RE-AIM evaluation questions could be applied to existing AHA programs and partnerships.

## Potential Application of RE-AIM Evaluation Questions

### No known CVD risk factors

The Alliance for a Healthier Generation (www.HealthierGeneration.org) is a joint initiative between the AHA and the William J. Clinton Foundation. The Alliance for a Healthier Generation was created to stop the nationwide increase in childhood obesity by 2010 and is taking bold, innovative steps to help all children live longer and healthier lives. The 4 strategic initiatives target industry, health care, schools, and children themselves.

**Reach**

How many schools, children, health care organizations, or food manufacturers participate in selected prevention activities compared to target (eg, attendance and distribution numbers for programs or materials available)? How many of the participating schools are in vulnerable communities with disparate rates of obesity, diabetes, and heart disease?

**Efficacy/Effectiveness**

What was the impact of programs on fitness and weight measures (preintervention and postintervention and compared to national trends)? Does the target population buy or use (or intend to buy or use) the products or services (eg, lunch, snack options) that promote a healthful lifestyle? How did beverage consumption change in the schools? Does the program efficacy vary by community demographics and resources?

**Adoption**

How many of the potential providers or stakeholders (eg, schools) provide the intervention components? How does this compare to the need and the goal? Is variability in providing components related to resource disparities in the community? What are the community resources for promoting a healthy lifestyle? How much are the available resources used? How did beverage policy change in the schools?

**Implementation**

What are the barriers to providing the targeted service? Are the programs developed consistent with the AHA and Alliance recommendations for a healthful lifestyle? How feasible is intervention implementation in a variety of real-world settings?

**Maintenance**

Are school and other programs sustainable based on annual reviews? Will the target population continue to use the facility (eg, path for walking), goods or services (eg, purchasing low-fat milk) after the program or campaign is over?

### Known or identified risk factors

The AHA does not provide direct services to people with cardiovascular risk factors. However, the AHA's Web site provides decision trees to guide health professionals in treating risk factors and self-help programs for people who have cardiovascular risk factors, for example, diabetes or hypertension (www.americanheart.org/presenter.jhtml?identifier=2114 and www.americanheart.org/presenter.jhtml?identifier=3044887).

**Reach**

How many people with the identified risk factor (diabetes or hypertension) use the Web site? How many health professionals accessed risk factor reduction information?

**Efficacy/Effectiveness**

How well did patient users achieve goals for a healthy lifestyle and reduce CVD risk status? Potential measures include self-reported control of CVD risk factors such as lipid profile, blood pressure, and blood glucose as well as measures such as body weight, dietary intake, and physical activity. Do rates of achieving goals differ?

**Adoption**

How do Web site users compare with the general population and the vulnerable subgroups with diabetes or hypertension? Are population groups with disparate rates of risk factors using the Web site? How do these health professionals compare to others in their profession?

**Implementation**

Which components of the programs were used? Were the Web site features used as planned? What are the barriers to implementing the recommendations for patients and health care providers? Are there more barriers for population groups with diabetes and hypertension health disparities? What is needed to make effective programs more translatable to the real world?

**Maintenance**

Will program users continue desired patient or self-care behaviors? Will Web site links continue to have sponsorship and be available on the AHA Web site?

### Acute CVD

At this risk stage, the focus shifts to immediate emergency care issues. The AHA has a national network of community-based programs designed to reduce response times to cardiac emergencies by improving access to automatic external defibrillators among laypeople. Success of these Operation Heartbeat programs depends in part on the public's knowledge of the warning signs of a myocardial infarction (MI) and the appropriate response to cardiac arrest victims (www.americanheart.org/presenter.jhtml?identifier=10000046&title=Operation Heartbeat).

**Reach**

What is the availability of acute cardiac services in the target community? What is the estimated need? How many people in the community know about or know how to use the service? How does the availability of service compare to the number of people who need the service?

**Efficacy/Effectiveness**

Do community residents receive needed treatment in a timely manner (eg, emergency response time)? Does response time vary based on community characteristics?

**Adoption**

How many public facilities employ staff who know how to use emergency services? How available are catheterization and revascularization services? Are some communities with excess morbidity and mortality lacking access to these services?

**Implementation**

What are the barriers to increasing access to emergency or cardiac services? Can the findings be generalized and translated to programs elsewhere? What resources and other factors are needed to do so?

**Maintenance**

How can a program continue to address service needs? Examination of program sustainability can be based on audits of emergency responses, and cardiac event mortality can be evaluated based on medical charts and review of emergency service logs.

### Rehabilitation

The AHA promotes a partnership between patients and their doctors, nurses, pharmacists, and other health care professionals to help patients change their health habits. Patients take an active role in making these changes (www.americanheart.org/presenter.jhtml?identifier=3047638 and www.americanheart.org/presenter.jhtml?identifier=4713).

**Reach**

How many people receive AHA-recommended rehabilitation services (eg, after having a heart attack or stroke)? What is the availability of cardiac rehabilitation in the target community? What is the estimated need? How many people in the community know about or know how to use the service? Are some communities lacking access to rehabilitation services?

**Efficacy/Effectiveness**

What is the overall quality of life and health for people who have had a CVD event? How are demographic and community characteristics related to health and quality-of-life measures?

**Adoption**

How many facilities provide recommended components of rehabilitation? How does the quality of rehabilitation facilities relate to disparities in outcomes?

**Implementation**

What are the barriers to offering comprehensive (including lifestyle counseling) rehabilitation services in underserved communities? Staff and patient interviews can provide insights about services offered and their use.

**Maintenance**

How well do patients maintain recommended lifestyle changes after participating in a rehabilitation program? What resources are institutionalized as ongoing rehabilitation services? Is a comprehensive rehabilitation program available over time?

### Chronic CVD

The AHA launched the Get With the Guidelines (GWTG) program in 2000 to help hospitals treat patients with evidence-based medicine known to improve health outcomes (www.americanheart.org/presenter.jhtml?identifier=3049656). The GWTG program has 3 modules: coronary artery disease, heart failure, and stroke. Each module addresses specific clinical practices and lifestyle changes.

**Reach**

How many hospitals participate in the GWTG program? How many patients are treated with the GWTG program? What is the estimated need? How many people in the community know about or know how to use the service? Do underserved communities have access to hospitals in the GWTC program?

**Efficacy/Effectiveness**

How do the statistics for second MI compare to rates where the GWTG program is not available? Do community residents receive treatment needed in a timely manner? How many stroke and MI patients receive treatment within the recommended time window? What are the disparities in the rates of secondary prevention of stroke and MI?

**Adoption**

What percentage of the staff and hospital programs follow the guidelines as recommended? Do GWTG hospitals in underserved communities implement guidelines at the same level as other hospitals?

**Implementation**

What are the barriers to secondary prevention? What are the views with regard to secondary prevention in patients, families, and health providers? Is there sufficient coordination to meet patients' needs? Can the findings be generalized and translated to programs elsewhere? What resources and other factors are needed to do so?

**Maintenance**

How many hospitals continue to meet the guidelines every year? Do hospitals in underserved communities continue to meet guidelines at the same rate as those in other communities?

## Practice Implications

The RE-AIM evaluation rubric provides a framework for examining AHA programs and collaborative projects. Applying the rubric to each of the identified stages of risk reduction can provide insights for addressing disparities. Creating innovative partnerships and enhancing community-based participatory research (community organizations, community clinics) will allow the AHA to increase healthy lifestyle educational programs and the number of community organizations that implement these programs. These efforts could lead to changes in policies, including access to care, physician training on cultural awareness, and treatment guidelines sensitive to ethnic differences.

## Acknowledgments

We thank the AHA Industry Nutrition Advisory Panel for providing an unrestricted grant to address the needs of special populations in the nutrition behavioral roundtable, which led to the development of this paper. We are especially grateful to JoAnn Hamamura, MS, CNS, who served as industry cochair for the special populations workgroup, and Vickie Peters, MS, who served as the AHA facilitator.

## Appendix. Potential Intervention Issues at Each Stage of Risk

### Population with no known risk factor** (may not know risk status)**

**Factors to consider in risk assessment**

- Screening guidelines
- Priorities based on hierarchy of needs (eg, food, housing)
- Family history and genetic factors related to risk
- Lack of knowledge as a barrier
- Lack of access to care or diagnosis

**Life experiences related to disparity**

- Often negative with health institutions (racism or poverty)
- Values and beliefs (denial of illness)
- Environmental influences on habits
- Competing priorities relating to sense of self within cultural, ethnic, or sex groups
- Limited access to risk assessment and screening

**Intervention opportunities**

- Partnering with community programs, community activism
- US Department of Agriculture community-supported agriculture
- Social gathering places (churches, schools, malls, beauty shops)
- Sports and leisure-time activities
- Marketing strategies for partnering
- Housing units
- Community gatekeepers

### Known or identified risk factor (awareness of risk)

- Feeling vulnerable
- Traditional roles in family or culture (motivation to change)
- Women (care for family vs self)
- Acknowledge risk (partnering/knowledge/perception/beliefs)
- Awareness of demographic or social changes in communities
- Projections for health systems and needs must be timely
- Disparity between haves and have nots (disparity could increase as technology advances improve medication options and tailoring of intervention based on gene expression)
- Bioethical research (labeling concerns, rationing of services, tests, or procedures)
- Access to health care providers
- Enablers of choice
- How to process knowledge (need for an environment that supports it)

### Acute event (onset of symptoms leading to emergency care)

- Recognition of symptoms and knowing what action to take
- Appropriate care (timely, transcultural communications)
- Education in hospital to start or reinforce desired behaviors
- Guidance to patients (cultural issues)
- Bringing in family and educating family (impact of acute event on roles)
- Assessing lifestyle issues (cultural competency of providers)
- Planning for follow-up

### Rehabilitation

- Coronary heart disease (perception of patient and family, cultural issues)
- Peripheral vascular disease (perception of patient and family, cultural issues)
- Eating habits and nutrition (individual and cultural comfort foods, perceived role of nutrition and physical activity in determining health)
- Exercise program (barriers to implementing exercise at home)
- Focus on individual requires family support (role of individual within family)
- Access to rehabilitation services (distance, communication with staff)
- Predictors of access and use (within context of the model)
- Mental health issues (cultural aspects, fatalism)

### Secondary prevention

- Maintenance of rehabilitation learning and habits (sources of reinforcement and access)
- Traditional food habits (integration of dietary modifications)
- Motivation to change and perceived control to prevent recurrence
- Knowledge of how to change, adapt (sources of information)
- Programs not designed to reflect culture, income
- Cultural differences related to illness and recovery
- Attitudes about physical activity (individual and cultural)
- Diversity in style of positive coping

## References

[1] Mensah GA, Mokdad AL, Ford ES, Greenlund KJ, Croft JB (2005). State of disparities in cardiovascular health in the United States. Circulation.

[2] Cooper RS, Cutler J, Desvigne-Nickens P, Fortmann SP, Friedman L, Havlik R (2000). Trends and disparities in coronary heart disease, stroke, and other cardiovascular diseases in the United States: finding of the National Conference on Cardiovascular Disease Prevention. Circulation.

[3] Kaplan GA, Everson SA, Lynch JW, Smedley BD, Slyme SL (2000). The contribution of social and behavioral research to an understanding of the distribution of disease: a multilevel approach. Promoting health: intervention strategies from social and behavioral research.

[4] Swinburn B, Egger G, Raza R (1999). Dissecting obesogenic environments: the development and application of a framework for identifying and prioritizing environmental interventions. Prev Med.

[5] Nestle M, Jacobson MF (2000). Halting the obesity epidemic: a public health policy approach. Public Health Rep.

[6] Kumanyika SK, Obarzanek E, Stettler N, Bell R, Field AE, Fortmann SP (2008). Population-based obesity: the need for comprehensive promotion of healthful eating, physical activity, and energy balance. Circulation.

[7] Centers for Disease Control and Prevention (2003). Self-reported concern about food security associated with obesity — Washington, 1995–1999. MMWR Morb Mortal Wkly Rep.

[8] Drewnowski A, Specter SE (2004). Poverty and obesity: the role of energy density and energy costs. Am J Clin Nutr.

[9] Laraia BA, Siega-Riz AM, Evenson KR (2004). Self-reported overweight and obesity are not associated with concern about enough food among adults in New York and Louisiana. Prev Med.

[10] Leung MW, Yen IH, Minkler M (2004). Community-based participatory research: a promising approach for increasing epidemiology relevance in the 21st century. Int J Epidemiol.

[11] Kaplan SA, Dillman KN, Calman NS, Billings J (2004). Opening doors and building capacity: employing a community-based approach to surveying. J Urban Health.

[12] Glasgow R, Lichtenstein E, Marcus AC (2003). Why don't we see more translation of health promotion research into practice? Rethinking the efficacy-to-effectiveness transition. Am J Public Health.

